# Role of the membrane potential in mitochondrial protein unfolding and import

**DOI:** 10.1038/s41598-019-44152-z

**Published:** 2019-05-21

**Authors:** Takehiro K. Sato, Shin Kawano, Toshiya Endo

**Affiliations:** 1 Department of Chemistry, Graduate School of Science, Nagoya University, Chikusa-ku, Nagoya 464-8602 Japan; 2 Faculty of Life Sciences, Kyoto Sangyo University, Kamigamo-motoyama, Kita-ku, Kyoto 603-8555 Japan; 3 Institute for Protein Dynamics, Kyoto Sangyo University, Kamigamo-motoyama, Kita-ku, Kyoto 603-8555 Japan; 4 Present Address: Spiber Inc. 234-1 Mizukami, Kakuganji, Tsuruoka, Yamagata 997-0052 Japan

**Keywords:** Mitochondria, Protein translocation

## Abstract

Newly synthesized mitochondrial precursor proteins have to become unfolded to cross the mitochondrial membranes. This unfolding is achieved primarily by mitochondrial Hsp70 (mtHsp70) for presequence-containing precursor proteins. However, the membrane potential across the inner membrane (ΔΨ) could also contribute to unfolding of short-presequence containing mitochondrial precursor proteins. Here we investigated the role of ΔΨ in mitochondrial protein unfolding and import. We found that the effects of mutations in the presequence on import rates are correlated well with the hydrophobicity or ability to interact with import motor components including mtHsp70, but not with ΔΨ (negative inside). A spontaneously unfolded precursor protein with a short presequence is therefore trapped by motor components including mtHsp70, but not ΔΨ, which could cause global unfolding of the precursor protein. Instead, ΔΨ may contribute the precursor unfolding by holding the presequence at the inner membrane for trapping of the unfolded species by the import motor system.

## Introduction

Proteins are programmed to attain a folded functional structure. However, transient unfolding of proteins is also required in essential cellular processes including translocation across biological membranes and selective degradation by ATP-dependent proteases^1,2^. Machineries involved in these processes have therefore activities to unravel folded protein substrates with the aid of external energy input.

About 70% of mitochondrial precursor proteins are synthesized with an N-terminal presequence, which guides the protein to mitochondria and is proteolytically processed upon import^3^. They cross the outer membrane through the TOM complex and the inner membrane through the TIM23 complex to reach the matrix. However, only the TIM23 complex can drive unfolding of even tightly folded protein domains since it is equipped with an import motor assembly composed of mitochondrial Hsp70 (mtHsp70; Ssc1p in yeast) and its partner proteins that actively catalyze precursor unfolding^4–6^. Mitochondrial presequences vary in lengths from 6 to 93 residues^3^, and import rates are usually ≥10-fold higher for precursor proteins with a long presequence (>70 residues) than those with a short presequence (<50 residues)^7–12^. Since presequences do not take ordered structures in aqueous solution, the N-terminus of a long presequence can cross the outer and inner membranes to reach the matrix without unfolding of the folded mature domain^7,8^. Then the incoming N-terminal segment of the presequence is grasped by the first mtHsp70 molecule at the outlet of the TIM23 channel in the matrix^8,11,13^. Next, a second mtHsp70 molecule cooperates with the first mtHsp70 to overcome the rate-limiting step of the import reaction, active unfolding of the folded mature domain outside the mitochondria by either of the mechanisms, the power stroke and Brownian ratchet models^11,14,15^. In the power stroke model, the first mtHsp70 tethered to the outlet of the TIM23 channel undergoes a conformational change to generate a mechanical pulling force exerted on the precursor protein, which drives unfolding of the folded mature domain outside the mitochondria^14^. In the Brownian ratchet model, spontaneous local unfolding of the mature domain adjacent to the presequence allows translocation of the unfolded segment through the import channel by Brownian motions once the first mtHsp70 dissociates from the TIM23 complex^11,16^. Then in both models, binding of a second mtHsp70 molecule to the translocated segment in the matrix prevents backsliding and refolding, thereby leading to global unfolding of the mature domain outside the mitochondria. Turn-over of mtHsp70 requires ATP hydrolysis. Although the Brownian ratchet vs. power stroke issue has been a matter of longstanding debate for the mechanism of active unfolding of folded precursor proteins with a long presequence by mtHsp70, current evidence favors the Brownian ratchet model^16–18^.

**Figure 1:**
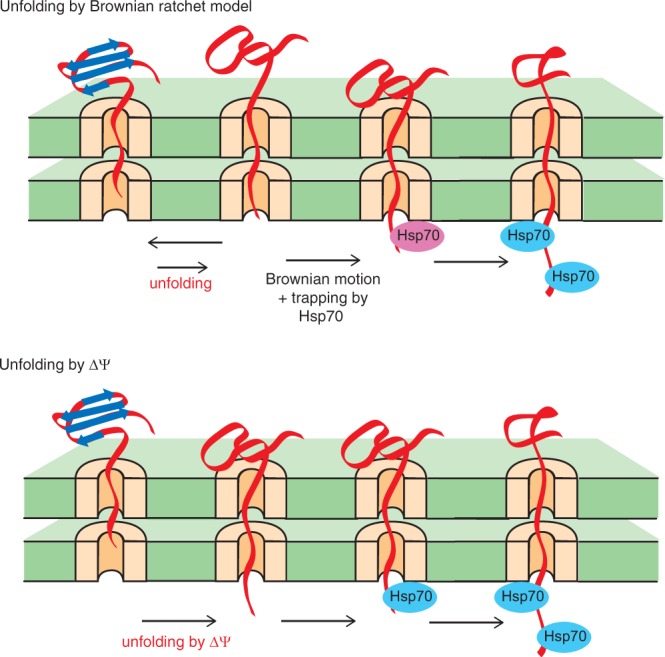
Unfolding of a short-presequence containing precursor protein by Brownian ratchet model (top) and by ΔΨ (bottom).

Although mtHsp70-facilitated active unfolding of precursor proteins with a long presequence (>70 residues) is efficient, the predominant length distribution of mitochondrial presequences does not actually fall in the range of a long presequence, but is 15–55 amino acid residues^3^. Nevertheless, a less efficient unfolding mechanism of precursor proteins with a short presequence (<50 residues) was not so extensively studied as that for long-presequence containing precursor proteins. For precursor proteins with a short presequence, the presequence is too short to cross the two membranes to interact with mtHsp70 in the matrix before unfolding of the mature domain, so that the unfolding limits the import rate^7,8^. The prevailing Brownian ratchet model suggests that a spontaneously unfolded species of the mature domain outside the mitochondria can be trapped by binding of a component of the TIM23 import system, such as a single mtHsp70 molecule in the matrix, to the incoming transiently unfolded segment that penetrates through the import channels by Brownian motions (Fig. 1, upper panel). This trapping can be sufficient to prevent retrograde movement of the protein and refolding outside the mitochondria, thereby leading to global unfolding of the mature domain^8,11^. The import rate is thus limited by the global unfolding of the domain outside the mitochondria coupled with the trapping of the unfolded species. Alternatively, the membrane potential across the inner membrane (ΔΨ; negative inside), which is required for the early step of translocation of presequence-containing precursor proteins across the inner membrane, that is engagement of the presequence with the TIM23 import channel and its gate opening^19,20^, could also drive active unfolding of precursor proteins with a positively charged short presequence by direct physical pulling at the level of the TIM23 complex^10^ (Fig. 1, lower panel). However, contribution of such ΔΨ-mediated direct unfolding to the global unfolding required for the import of precursor proteins remained unclear.

In the present study, we assessed the role of ΔΨ in unfolding of short-presequence containing precursor proteins for translocation across the inner membrane. For this purpose, we used fusion proteins consisting of the cytochrome *b*_2_ matrix-targeting signal or its variants followed by a folded mature domain. The cytochrome *b*_2_ matrix-targeting signal is unusual in that it contains a negatively charged residue Glu at position 15 in the middle of the targeting signal, which may antagonize the possible electrophoretic action of ΔΨ. We found that neutralization of the negative charge of Glu-15 in the cytochrome *b*_2_ presequence did not always lead to enhancement of the import rate of short-presequence containing fusion proteins, which is not consistent with the expectation for the ΔΨ-mediated unfolding. The effects of amino-acid replacements of residue 15 in the cytochrome *b*_2_ presequence on the import rate could be rather consistent with the previously analyzed peptide affinities for Hsp70 rather than electrostatic charges. On the basis of these findings, we propose a new role of ΔΨ in unfolding of short-presequence containing precursor proteins for translocation across the inner membrane.

## Results and Discussion

### Fusion proteins with an unoptimized short presequence can be imported into mitochondria inefficiently

Import rates are usually significantly higher for long-presequence containing precursor proteins than short-presequence containing ones^7–12^. This is because, while a long presequence can easily reach the matrix to bind to the first mtHsp70, thereby being prevented from disengagement from the TIM23 import system, a short presequence cannot reach mtHsp70 in the matrix before unfolding of the mature domain, resulting in inefficient binding to mtHsp70 and high tendency to slip off the TIM23 import channel^8,21^. However, Huang *et al*. observed that optimization of a short presequence could enhance the import rate significantly; the import rate of the barnase fusion protein with the short matrix-targeting signal (residues 1–32) of the cytochrome *b*_2_ presequence was ~10-fold increased by the Glu→Leu mutation at position 15 in the presequence^10^.

**Figure 2:**
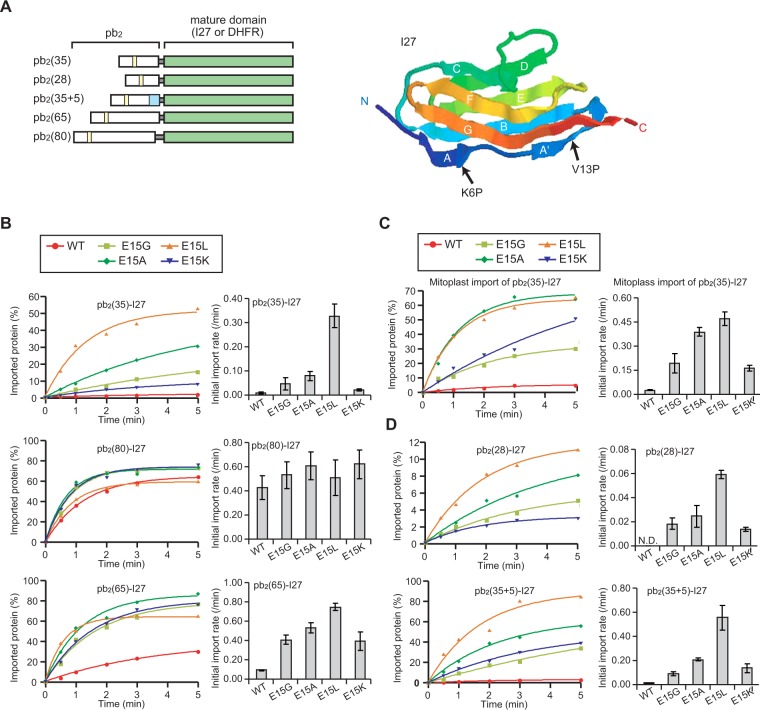
Mutations in the short presequences of the I27 fusion proteins increase import rates. (**A**) Left panel; schematic representation of the I27 and DHFR fusion proteins used in this study (except for Fig. 4). Arg-30 is replaced by Gly-30 and Leu-62 is replaced by Pro-62 in the first X residues of the cytochrome *b*_2_ presequence (pb_2_(X)); the R30G mutation eliminates the cleavage site for MPP in the matrix and the L62P mutation inactivates the intermembrane-space sorting signal in residues 36–65. Right panel; the structure of the I27 domain is shown with β-strands in ribbon model. The atomic coordinates were taken from Protein Data Bank (1TIT). (**B**) *In vitro* import of the radiolabeled pb_2_(35)-I27, pb_2_(80)-I27, and pb_2_(65)-I27 fusion proteins into mitochondria. The fusion proteins with the I27 domain (WT) or with its variants bearing a mutation E15G, E15A, E15L or E15K were incubated with isolated mitochondria for various time at 25 °C. Proteinase K (PK)-protected fractions were quantified and plotted against time (left panels). The amounts of radiolabeled proteins added to each reaction are set to 100%. Import rates (initial slopes of the import reactions) were plotted (right panels). Values are means ± SD. (**C**) *In vitro* import of the radiolabeled pb_2_(35)-I27 fusion proteins into mitoplasts with disrupted outer membranes^34^ was performed as in (A). (**D**) *In vitro* import of the radiolabeled pb_2_(28)-I27 and pb_2_(35 + 5)-I27 fusion proteins into mitochondria was performed as in (A). N.D., not determined.

To analyze the mechanism of the import-rate enhancement of short-presequence containing precursor proteins, we constructed model precursor proteins consisting of the segments containing matrix-targeting signal (residues 1–32) of the cytochrome *b*_2_ presequence fused to the 27th immunoglobulin (I27) domain of titin, which consists of two face-to-face β-sheets and was extensively studied as a passenger protein for unfolding pathways during mitochondrial protein import^12^. Thus, radiolabeled pb_2_(35)-I27, pb_2_(65)-I27 and pb_2_(80)-I27, fusion proteins of the matrix-targeting N-terminal 35 residues (pb_2_(35)), 65 residues (pb_2_(65)) and 80 residues (pb_2_(80)) of cytochrome *b*_2_ presequence followed by I27 (Fig. 2A, left panel), were synthesized in reticulocyte lysate and incubated with isolated yeast mitochondria. Protease-protected, imported fractions were quantified, and import rates were obtained. The import rate of pb_2_(35)-I27 with a short presequence was ~40-fold lower than that of pb_2_(80)-I27 with a long presequence as expected (Fig. 2B). Evidently, the I27 domain is tightly folded, so that the short pb_2_(35) presequence cannot cross the outer and inner membranes to reach the matrix for engagement with mtHsp70 without infrequent spontaneous unfolding of the I27 domain.

### Optimization of the short presequence for efficient import does not rely on electrostatic interactions with ΔΨ

Next we replaced negatively charged Glu-15 in the pb_2_(35) presequence with Leu to make pb_2_(35)E15L-I27 (Fig. 2A, left panel). The import rate of pb_2_(35)E15L-I27 with the E15L mutation was ~40-fold higher than that of pb_2_(35)-I27, as previously observed for barnase fusion proteins^10^, and was comparable with that of pb_2_(80)-I27 with a long presequence (Fig. 2B). The increased import rate due to the Glu→Leu substitution was previously ascribed to the enhanced interaction of the presequence with ΔΨ by the removal of a negative charge at residue 15, which could accelerate the possible ΔΨ-mediated unfolding^10^. However, Glu is unfavorable for binding to mtHsp70^17,18^, as well, and therefore its substitution by Leu may well promote stable trapping of the presequence by the mtHsp70 motor assembly. Stable trapping of the incoming presequence by a single mtHsp70 molecule in the matrix, after spontaneous N-terminal unfolding of the mature domain followed by the Brownian movement of the unfolded segments through the import channels, may potentially prevent refolding of the mature domain, thereby leading to its global unfolding.

We thus examined the effects of replacement of Glu-15 with neutral Gly or Ala, or with positively charged Lys on import, and found that the import rates became only 6-fold, 10-fold, and 3-fold higher, respectively, than that of pb_2_(35)-I27 (Fig. 2B). Note that residue 15 is located at the border the hydrophilic and hydrophobic sides upon formation of the amphiphilic helix for receptor recognition, so that the charge at this position may affect the very early step of the import, i.e. recognition by the receptor Tom20 and/or Tom22. However, under the present condition of the presence of a functional excess of mitochondria, binding of precursor proteins to receptors is unlikely to be rate-limiting^22,23^. These results suggest that the net charge at position 15 is not crucial for enhancement of the import rates of the pb_2_(35)-I27 mutants. Rather, enhancement of the import rates by different amino-acid replacement of residue 15 follows the order of hydrophobicity^24^ and is thus correlated well with the amino-acid preference (Leu > Ala > Gly, Lys, Glu) in short (~7-residue) peptides for binding to Hsp70 as unfolded substrates^25–28^. Therefore, the enhanced unfolding is likely due to increased efficiency in trapping of the pb_2_(35) presequence mutants by ratchet components of the TIM23 machinery such as mtHsp70 through hydrophobic interactions.

### Optimization of the short presequence for efficient import is sensitive to its position at the inner membrane

Although the import rate was markedly higher for pb_2_(80)-I27 with a long presequence than for pb_2_(35)-I27, mutations of E15L, E15A, and E15K in the pb_2_(80) presequence did not significantly affect the import rates of the pb_2_(80)-I27 fusion proteins (Fig. 2B). This suggests that the import rates have reached the maximal import-rate regime, where unfolding of the I27 domain is so fast that turnover of the mtHsp70 import motor rather than unfolding becomes rate limiting^7^. pb_2_(65)-I27 fusion proteins with a medium length (65 residues) of the presequence behave between the pb_2_(35)-I27 and pb_2_(80)-I27 fusion proteins in import into mitochondria (Fig. 2B). This is consistent with the geometrical constraint that the segment around residue 15 in pb_2_(65)-I27 fusion proteins (51-residue N-terminal to the folded I27 domain) can be close the outlet of the TIM23 channel^9,10,29,30^ and small conformational fluctuations of the I27 domain may well render residue 15 accessible to the motor component like mtHsp70 for grasp in the matrix^8,13^.

If binding efficiency for the motor component is not so low, a prediction stemming from those results is that import into mitoplasts, where the outer membrane is disrupted by osmotic swelling, makes the geometrical situation of pb_2_(35)-I27 fusion proteins closer to that of pb_2_(65)-I27 fusion proteins in import into mitochondria. Indeed, the effects of amino-acid replacement of residue 15 of pb_2_(35)-I27 fusion proteins on import into mitoplasts (Fig. 2C) became similar to those on import of pb_2_(65)-I27 fusion proteins into mitochondria rather than those on import of pb_2_(35)-I27 fusion proteins into mitochondria (Fig. 2B). For example, the import rate of pb_2_(35)E15A-I27 was nearly the same as that of pb_2_(35)E15L-I27 for mitoplasts (Fig. 1C), whereas pb_2_(35)E15L-I27 was imported into mitochondria ~4-fold faster than pb_2_(35)E15A-I27 (Fig. 2B).

To further rule out the possibility of the active role of ΔΨ in the effects of amino-acid replacement of residue 15 on import rates, we changed the distance between the mutation position and the folded I27 domain of the pb_2_(35)-I27 fusion proteins because ΔΨ is effectively limited to the portion of the import channel in the inner membrane^10,31^. When mutations were advanced by 5 residues (pb_2_(35 + 5)-I27 fusion proteins) or receded by 7 residues (pb_2_(28)-I27 fusion proteins) in the presequence from the I27 domain (Fig. 2A), relative effects of mutations of E15G, E15L, E15A and E15K on import remain nearly the same, although overall import rates became higher with increasing lengths of the presequences (Fig. 2D). Insensitivity to the positions of the mutations supports the interpretation that the enhanced import rate or unfolding by the E15L (and E15A) mutation cannot be ascribed to increased interactions of the presequence with position-sensitive ΔΨ. Therefore, the enhanced unfolding of the short-presequence containing I27 fusion proteins is not driven by the mechanical pulling by ΔΨ, but by enhanced binding of the presequence to the TIM23 import motor system including a single mtHsp70 molecule. For example, the mtHsp70 molecule could trap the N-terminally unfolded species of the precursor protein stably in the matrix, thereby inducing global unfolding without cooperation with the second mtHsp70 molecule.

### Optimization of the short presequence for efficient import is not affected by stability of the mature domain

**Figure 3:**
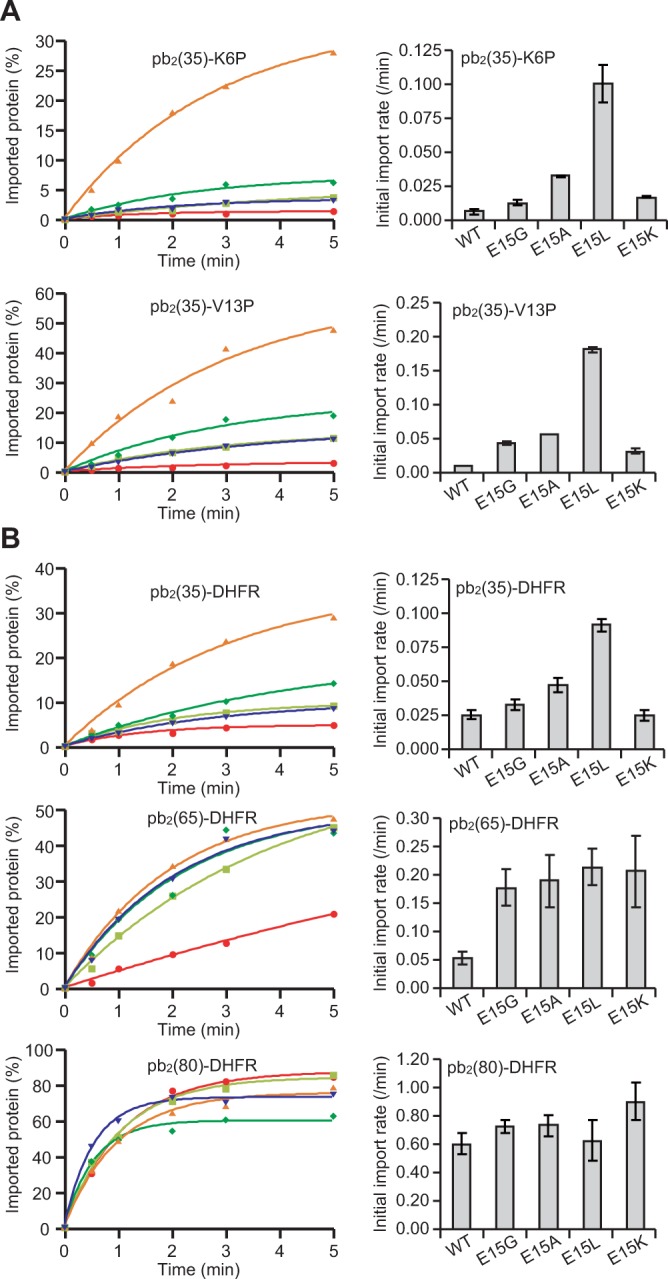
Effects of mutations in the short presequences of the fusion proteins with different mature domains. *In vitro* import of the radiolabeled pb_2_(35)-I27^K6P^ and pb_2_(35)-I27^V13P^ fusion proteins (**A**) and pb_2_(35)-DHFR, pb_2_(65)-DHFR, and pb_2_(80)-DHFR fusion proteins (**B**) into mitochondria was performed as in Fig. 1B.

If mutations in the presequence only affects the trapping efficiency of the spontaneously unfolded species of the precursor protein, changes in stabilities of the folded mature domains will not affect the relative effects of the mutations in the presequence on import rates. We thus tested the import of fusion proteins with I27^K6P^ and I27^V13P^, the I27 variants with Lys→Pro substitution at residue 6 and Val→Pro substitution at residue 13, respectively, which cause different effects on the I27 structural stability^12^ (Fig. 2A, right panel). Titin I27 consists of 8 β-strands, short A and A’ strands and long B, C, D, E, F, and G strands from the N- to C-termini. The K6P mutation in the most N-terminal short β-strand A increases the mechanical stability of the A- and B-strand β-sheet pair, resulting in the decreased import rates of short-presequence containing I27 fusion proteins^12^. The V13P mutation in the second N-terminal short β-strand A’ decreases the mechanical stability of the A’- and G-strand β-sheet pair, which, however, does not affect the import rates of short-presequence containing I27 fusion proteins since once the A strand is spontaneously detached from the B strand, unfolding of the rest of the molecule, including detachment of the A’ strand from the G strand, follows immediately^12^. Now the relative effects of mutations at position15 in the cytochrome *b*_2_ presequence were similar between those for a series of the I27^K6P^ fusion proteins and for a series of the I27^V13P^ fusion proteins (Fig. 3A).

We also tested the fusion proteins with mouse dihydrofolate reductase (DHFR) instead of I27 as a mature domain for different lengths of the cytochrome *b*_2_ presequences. In contrast to I27, which spontaneously undergoes local unfolding around the N-terminal region by transient detachment of the strand A and B β-sheet pair, since both the N-terminal and C-terminal segments of DHFR are involved in the same β-sheet structure and the N-terminal β-strand of DHFR is further sandwiched by the two α-helices, DHFR spontaneously undergoes only global unfolding, not local unfolding around the N-terminal region^18^. Relative effects of mutations at position15 in the presequence were similar between a series of DHFR fusion proteins (Fig. 3B) and a series of I27 proteins (Fig. 2B) irrespective of the lengths of the presequences, yet import of the pb_2_(35)-DHFR fusion proteins was less sensitive to the mutations at residue 15. This is likely because transient global unfolding of the DHFR domain would allow a longer unfolded segment with downstream mtHsp70 binding sites to enter the matrix than the I27 fusion proteins, which may well suppress the effects of mtHsp70 avoidance residue at position 15.

Taken together, mutations at position15 in the cytochrome *b*_2_ presequence are apparently not sensitive to the changes in stabilities or structures of the folded mature domains like I27 variants and DHFR. This strongly suggests that the presequence primarily affects the trapping efficiency of the spontaneously unfolded precursor proteins.

### Retardation of the presequence cleavage could reflect mtHsp70 binding to the presequence

**Figure 4:**
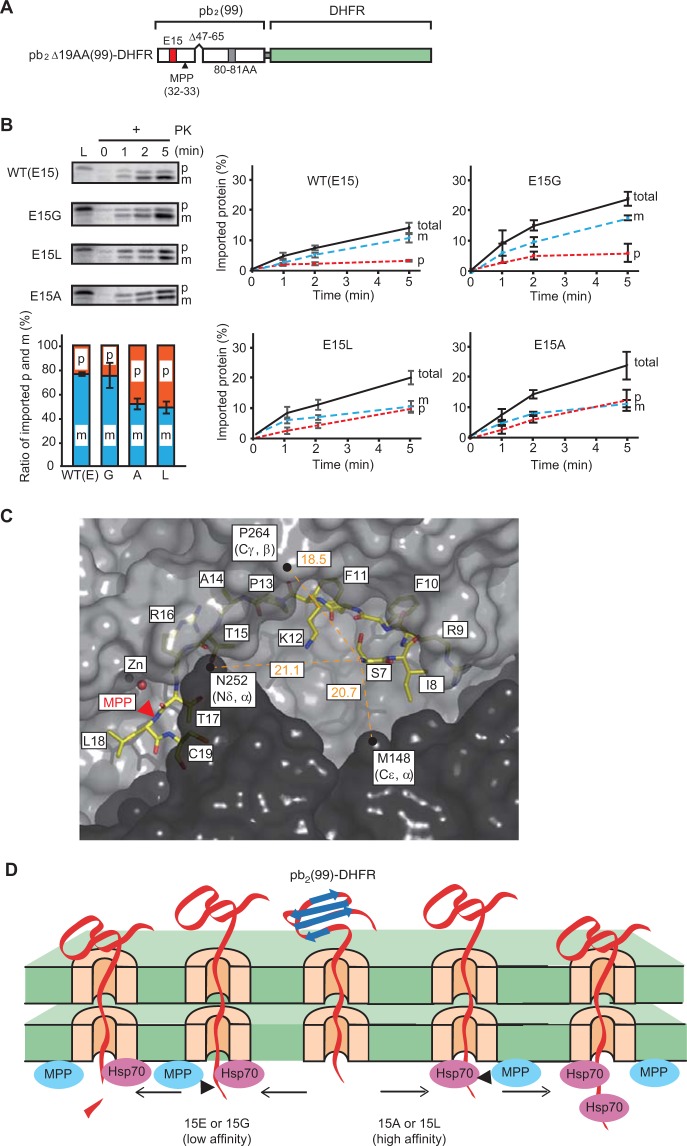
Competition between the motor-component binding to presequence and presequence cleavage by MPP. (**A**) Schematic representation of pb_2_Δ19AA(99)-DHFR and its derivatives used for import and MPP cleavage analyses. The first 99 residues of the cytochrome *b*_2_ precursor (the 80-residue presequence with the deletion of residues 47–65 in the intermembrane space sorting signal and with the ^80^N^81^E **→** ^80^A^81^A mutation at the second processing site, plus 19 residues of the mature protein) was attached to the N-terminus of DHFR. With these mutations, pb_2_Δ19AA(99)-DHFR fusion proteins are targeted to the matrix and do not receive the second processing of the presequence by Imp1^30,35^. (**B**) *In vitro* import of the radiolabeled pb_2_Δ19AA(99)-DHFR fusion proteins into mitochondria. The fusion protein (WT) or its variants bearing a mutation E15G, E15A, or E15L were incubated with isolated mitochondria for various time at 25 °C, and proteinase K (PK)-protected fractions (upper left panels) were quantified and plotted against time (central and right panels). L, 5% (WT and E15L) or 10% (E15G and E15A) of the input as loading controls. The amounts of radiolabeled proteins added to each reaction are set to 100%. The amounts of the imported proteins with (m) and without (p) the MPP cleavage at 5 min import were plotted (lower left panel, the total amounts (m + p) are set to 100%). Values are means ± SD. Full-length gel images are presented in Supplementary Fig. S1. (**C**) The crystal structures of the cleavage-deficient mutant of yeast MPP in a complex with the cytochrome oxidase subunit IV presequence peptide (1HR8). Two subunits of the MPP homo-dimer are represented by their surface diagram (dark gray for subunit a and light gray for subunit b) and the peptide (residues 7–18) drawn in stick form. Residues 1–6 are not visible. MPP cleavage site of the peptide between residues 17 and 18 are shown by the red arrowhead. The distances (Å) between S7 (N_α_) of the peptide and the MPP residues at the entrance of the dimer interface (N_δ_ of N252 of subunit b, C_γ_ of P264 of subunit b, and C_ε_ of M148 in subunit a) are indicated in orange. The N-terminal residue of the peptide corresponds to position -17 (from the MPP cleavage site) and is supposed to be close to the entrance. This panel suggests that MPP would sterically compete with mtHsp70 in binding to residue 15 of the pb_2_ presequence. (**D**) Model of competition between presequence cleavage by MPP and binding by mtHsp70 in the matrix. In this figure, mtHsp70 was assumed to be a presequence-binding protein of the import motor system for simplicity.

~65 residues^30^ or ≥52 residues^8^ in front of the folded DHFR domain on the cytosolic side of mitochondria were found to be sufficient to span both mitochondrial membranes and to allow tight binding of mtHsp70 to the segment exposed into the matrix. We thus designed matrix-targeted pb_2_Δ19AA(99)-DHFR fusion proteins, in which residue 15 is positioned 65 residues N-terminal to the folded DHFR domain (Fig. 4A). When we imported pb_2_Δ19AA(99)-DHFR (WT) or its derivatives with the E15G, E15A, or E15L mutation, cleavage of the presequence by mitochondrial processing peptidase (MPP) was significantly retarded for the E15A and E15L mutants as compared with the E15G mutant or WT protein (Fig. 4B). Since residue 15 (position -18) is 18 residues apart from the MPP cleavage site (between residues 32 and 33) and from the MPP recognition site (positions −2 and +1 from the cleavage site)^3,32^, the observed retardation effects are unlikely due to an altered substrate specificity for MPP. Rather, tight binding of mtHsp70 and/or other motor components to the matrix-exposed segment with the E15A or E15L mutation may sterically hinder the access of MPP to the presequence cleavage site (Fig. 4C). Such a competition between the motor component binding to and MPP cleavage of the presequence for the E15A and E15L mutants is consistent with the interpretation that amino-acid substitution for residue 15 affects the trapping efficiency of the presequence by motor components in mitochondria (Fig. 4D).

### ΔΨ facilitates initial binding of mtHsp70 to short presequences

**Figure 5:**
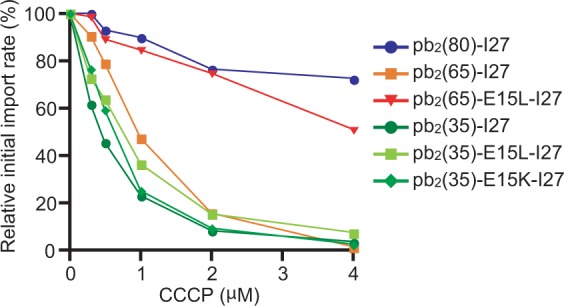
Import of short-presequence containing I27 fusion proteins is susceptible to reduction of ΔΨ. *In vitro* import of the radiolabeled pb_2_(35)-I27, pb_2_(80)-I27, and pb_2_(65)-I27 fusion proteins into mitochondria at 25 °C that were pre-treated with various concentrations of CCCP. PK-protected fractions were quantified and initial import rates were plotted against CCCP concentrations. Initial import rates for mitochondria without CCCP treatment are set to 100%.

**Figure 6:**
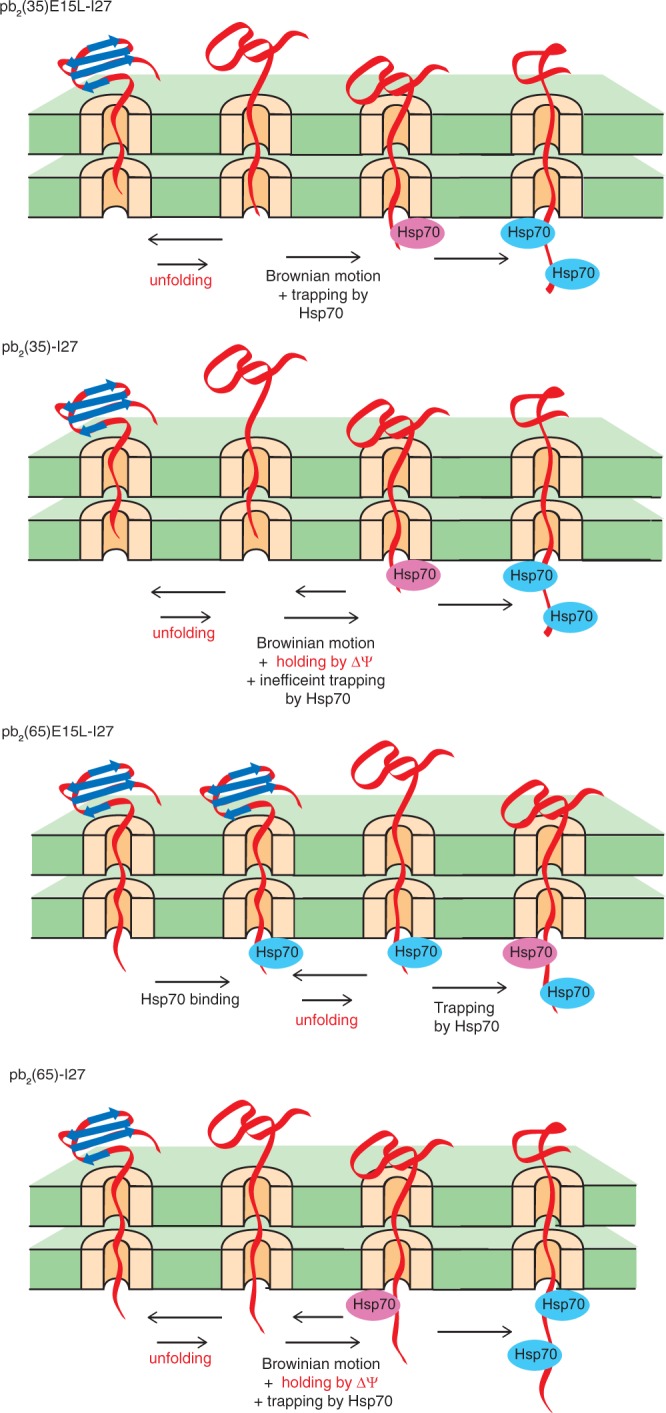
Proposed Brownian ratchet model for unfolding of short-presequence and medium-presequence containing precursor proteins. The Brownian ratchet model for unfolding of precursor proteins with a short preseuqnece, pb_2_(35)E15L-I27 and pb_2_(35)-I27, and precursor proteins with a medium-length presequence, pb_2_(65)E15L-I27 and pb_2_(65)-I27, are shown. In this figure, mtHsp70 was assumed to be an only presequence-binding protein of the import motor system for simplicity. The Hsp70 molecule that traps the unfolded species and contribute to active unfolding is shown by a pink oval.

Does ΔΨ play no role in the Brownian-ratchet unfolding of the short-presequence containing precursor proteins driven by a single mtHsp70 molecule? To address this question, we lowered ΔΨ of mitochondria prior to import by adding increasing concentrations of a protonophore, carbonyl cyanide *m*-chlorophenylhydrazone (CCCP). Import of pb_2_(80)-I27 with a long presequence hardly depended on the present concentration range of CCCP, suggesting that ΔΨ is not crucial for the I27 domain unfolding if the presequence is sufficiently long (Fig. 5). On the other hand, import rates of pb_2_(35)-I27, pb_2_(35)E15L-I27 and pb_2_(35)E15K-I27 with a short presequence decreased significantly with increasing concentrations of CCCP, pointing to the contribution of ΔΨ to the I27 domain unfolding when the presequence is short. Since import rates of both pb_2_(35)-I27 and pb_2_(35)E15L-I27 were sensitive to CCCP concentrations, ΔΨ should not primarily contribute to the enhancement of rate-limiting unfolding of the I27 domain by the E15L mutation. Rather, ΔΨ is important for both pb_2_(35)-I27 and pb_2_(35)E15L-I27, irrespective of their unfolding efficiencies. Possible explanation is that ΔΨ could contribute to holding the short presequence at the level of the inner membrane to suppress slippage of the precursor protein, but only less efficiently than mtHsp70 directly acting on pb_2_(80)-I27 fusion proteins (Fig. 6). This ΔΨ-facilitated holding of the short presequence at the inner membrane is especially important for inefficient binding of motor components like mtHsp70 to the wild-type pb_2_(35) presequence.

Consistently with this interpretation, the import rate of pb_2_(65)-I27, but not of pb_2_(65)E15L-I27, was also sensitive to reduction of ΔΨ. This suggests that the higher affinity of the pb_2_(65)E15L presequence for e.g. mtHsp70 than the wild-type pb_2_(65) presequence allows mtHsp70 to substitute for ΔΨ in holding of the presequence to prevent its slippage off the TIM23 complex, resulting in the switch from the Brownian ratchet by a single mtHsp70 molecule to the one by cooperation of two mtHsp70 molecules (Fig. 6).

Of note, the role of mtHsp70 in trapping of the short presequence that leads to global unfolding of precursor proteins can be tested by using yeast mtHsp70 mutant strains. The *ssc1–2* mutation in mtHsp70 does not impair substrate binding ability of mtHsp70, but leads to defective turnover of mtHsp70 in binding to Tim44, a mtHsp70 anchoring site of the TIM23 complex^15,33^. Huang *et al*. found that import of the pb_2_(35)E15L fusion protein was not affected by the *ssc1–2* mutation while the *ssc1-2* mutation strongly impaired import of the pb_2_(95) fusion protein, which requires at least two mtHsp70 molecules operating in a hand-over-hand manner^10^; the first Hsp70 must dissociate from the TIM23 complex before second mtHsp70 grasp the unfolded segment^8,11,13^. Therefore long-presequence containing precursor proteins are unfolded by trapping by multiple mtHsp70 molecules, but do not require ΔΨ for unfolding, while short-presequence containing precursor proteins require only the trapping by a single mtHsp70 molecule, which is facilitated by holding of the presequence at the level of the inner membrane by ΔΨ.

## Conclusions

In the present study, we demonstrated that the primary act of ΔΨ on positively charged presequences does not unravel folded mature domains of short-presequence containing precursor proteins. Rather, the folded mature domains are unraveled by spontaneous unfolding followed by stable trapping by a single mtHsp70 molecule (Fig. 6). The efficiency of this unfolding of the mature domain is, if the presequence is optimized, comparable to that of the two mtHsp70 molecule-driven unfolding of long-presequence containing precursor proteins. These results collectively suggest that the Brownian ratchet can explain active unfolding of precursor proteins with a short presequence by mtHsp70 molecules (and/or other TIM23 import system components) for protein import. In other words, the amplitude and frequency of spontaneous N-terminal unfolding of the mature domain are sufficient, if harvested efficiently, to promote rate-limiting global unfolding of the mature domain, irrespective of the lengths of the presequences.

The present results do not rule out the contribution of ΔΨ, if not primarily, in unfolding of mitochondrial precursor proteins. Indeed, ΔΨ likely contributes to mtHsp70 trapping of spontaneously unfolded species of precursor proteins by holding of the short presequence at the level of the inner membrane to suppress slippage of the precursor protein (Fig. 6). Since the presequences of authentic mitochondrial precursor proteins may have evolved to match the folding and translocation characteristics of the mature domains, contribution of ΔΨ and the mtHsp70 import motor could differ for different precursor proteins. For example, the recently discovered pathway of insertion of N-anchor outer-membrane proteins from the intermembrane side requires ΔΨ and the TIM23 complex, yet the ΔΨ requirement is bypassed for truncated substrate proteins^34,35^, and tightly folded domains block this pathway (unpublished results). This may suggest that ΔΨ may contribute to unfolding of loosely folded substrate proteins for translocation across the outer membrane perhaps by the Brownian ratchet mechanism or electrophoretic effects, although its unfolding activity is only weak. The Brownian ratchet mechanism with trapping of spontaneously unfolded species by various components including mtHsp70 appears to be a sufficient and minimal mechanism to achieve active unfolding of precursor proteins in mitochondrial protein import.

## Materials and Methods

### Model fusion proteins

The I27 fusion proteins (Fig. 2A, left panel) used in this study were made as follows. The genes for pb_2_(35)-I27 and pb_2_(80)-I27 carrying the R30G and L62P mutations were made in the previous study^12^, and pb_2_(28)-I27, p (35 + 5)-I27 and pb_2_(65)-I27 were derived from pb_2_(80)-I27 by PCR. The I27^K6P^ fusion protein was derived by PCR from pb_2_(35)-K6P used previously^12^. The genes for the DHFR fusion proteins were constructed by using genes for pb_2_-I27 fusion proteins^12^ and pb_2_(220)-DHFR or its derivatives^36^. Then point mutations (E15G, E15A, E15L and E15K) in the presequences were introduced into the genes for those fusion proteins.

### *In vitro* Import

*In vitro* protein import into isolated mitochondria was performed as described previously^37^. Briefly, the fusion proteins were synthesized in rabbit reticulocyte lysate by coupled transcription/translation in the presence of ^35^S-methionine. The radiolabeled fusion proteins were incubated with isolated yeast mitochondria (0.5 mg protein/ml) in import buffer (250 mM sucrose, 10 mM MOPS-KOH, pH 7.2, 80 mM KCl, 2.5 mM KPi, 2 mM methionine, 5 mM dithiothreitol, 5 mM MgCl_2_, 2 mM ATP, 2 mM NADH, 1% BSA) at 25 °C. The import reactions were stopped by adding valinomycin to 10 µg/ml. Each sample was halved and one aliquot was treated with 100 µg/ml proteinase K (PK) for 30 min on ice. After addition of 1 mM phenylmethylsulfonyl fluoride, the mitochondria were reisolated by centrifugation, and were washed once with SEM buffer (250 mM sucrose, 5 mM EDTA, and 10 mM MOPS-KOH, pH 7.2). Proteins were analyzed by SDS-PAGE and radioimaging with a Storm 860 image analyzer (Amersham Biosciences). CCCP treatment was performed as described previously^10^.

## Supplementary information

Fig. S1
