# Supplementary material for: Role of the membrane potential in mitochondrial protein unfolding and import
Source: Sci Rep. 2019 May 21;9:7637. doi: 10.1038/s41598-019-44152-z (PMC6529458; doi:10.1038/s41598-019-44152-z)
Supplement: Supplementary file 1 — Fig. S1 [file 41598_2019_44152_MOESM1_ESM.pdf]

(Revised version)
Supplementary Information

Role of the membrane potential in mitochondrial protein unfolding and import

Takehiro K. Sato, Shin Kawano, & Toshiya Endo

Supplementary Figures S1

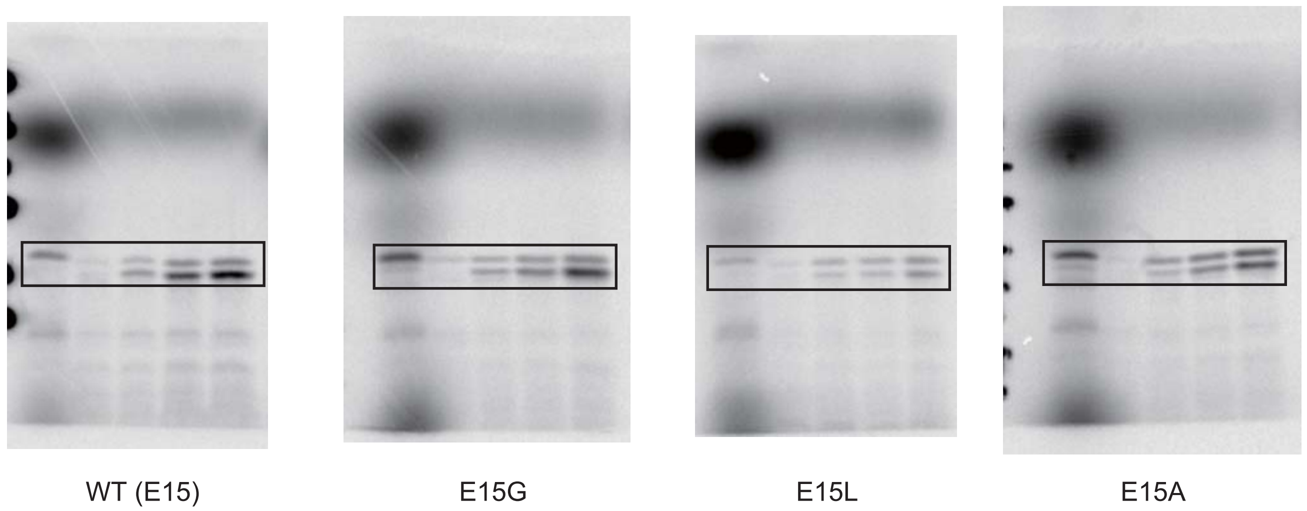

Figure S1. Full-length gel images for Fig. 4B
